# Diabetic retinopathy predicts cardiovascular disease independently of subclinical atherosclerosis in individuals with type 2 diabetes: A prospective cohort study

**DOI:** 10.3389/fcvm.2022.945421

**Published:** 2022-11-03

**Authors:** Esmeralda Castelblanco, Minerva Granado-Casas, Marta Hernández, Montserrat Pinyol, Eudald Correig, Josep Julve, Marina Idalia Rojo-López, Núria Alonso, Angelo Avogaro, Emilio Ortega, Didac Mauricio

**Affiliations:** ^1^Division of Endocrinology, Metabolism and Lipid Research, Washington University School of Medicine in St. Louis, St. Louis, MO, United States; ^2^DAP-Cat Group, Unitat de Suport a la Recerca Barcelona, Fundació Institut Universitari per a la Recerca a l’Atenció Primària de Salut Jordi Gol i Gurina, Barcelona, Spain; ^3^Center for Biomedical Research on Diabetes and Associated Metabolic Diseases (CIBERDEM), Instituto de Salud Carlos III, Barcelona, Spain; ^4^Department of Endocrinology and Nutrition, Hospital de la Santa Creu i Sant Pau and Sant Pau Biomedical Research Institute (IIB Sant Pau), Barcelona, Spain; ^5^Lleida Institute for Biomedical Research Dr. Pifarré Foundation IRBLleida, University of Lleida, Lleida, Spain; ^6^Department of Endocrinology and Nutrition, University Hospital Arnau de Vilanova, Lleida, Spain; ^7^Consorcio de Atención Primaria del Eixample (CAPSE), Grup Transversal de Recerca en Atenció Primària, Institut d’Investigacions Biomédiques August Pi i Sunyer (IDIBAPS), Barcelona, Spain; ^8^Department of Biostatistics, Universitat Rovira i Virgili, Reus, Spain; ^9^Department of Endocrinology and Nutrition, Germans Trias i Pujol Hospital and Research Institute, Universitat Autònoma de Barcelona, Badalona, Spain; ^10^Department of Medicine, Università di Padova, Padua, Italy; ^11^Diabetes Unit, Department of Endocrinology and Nutrition, Hospital Clínic de Barcelona, Barcelona, Spain; ^12^Center for Biomedical Research on Pathophysiology of Obesity and Nutrition (CIBEROBN), Instituto de Salud Carlos III (ISCIII), Barcelona, Spain; ^13^Institut d’Investigacions Biomèdiques August Pi i Sunyer, Barcelona, Spain; ^14^Faculty of Medicine, University of Vic (UVic-UCC), Barcelona, Spain

**Keywords:** type 2 diabetes, diabetic retinopathy, subclinical atherosclerosis, cardiovascular disease, major adverse cardiovascular events

## Abstract

**Background:**

Diabetic retinopathy (DR) and preclinical atherosclerosis are associated with higher cardiovascular risk. However, no studies have investigated the predictive role of DR and preclinical atherosclerosis jointly on cardiovascular events in subjects with type 2 diabetes (T2D). We aimed to assess the contribution of DR and subclinical atherosclerosis on the risk of adverse cardiovascular events in subjects with T2D without previous cardiovascular disease (CVD).

**Methods:**

We included two prospective cohorts of subjects with T2D from the same geographical area. Assessment of subclinical atherosclerosis was performed by carotid ultrasound. An ophthalmologist classified DR according to standard criteria. Cardiovascular outcomes considered for analysis were the following: ischemic heart disease, stroke, heart failure, peripheral artery disease, revascularization procedures, and cardiovascular mortality. Bivariable and multivariable predictive models were performed.

**Results:**

From a total of 374 subjects with T2D 44 developed cardiovascular events during the 7.1 years of follow-up. Diabetes duration, total cholesterol, and glycated hemoglobin (HbA1c) at baseline were higher in subjects who developed cardiovascular outcomes (*p* < 0.001, *p* = 0.026, and *p* = 0.040, respectively). Compared with subjects without events, those developing cardiovascular events had higher prevalence of retinopathy (65.9% vs. 38.8%, *p* = 0.001; respectively) and more than mild retinopathy (43.2% vs. 31.8%, *p* = 0.002; respectively). Furthermore, all-cause mortality was higher in subjects with MACE than those without events (13.6% vs. 3.3%, *p* = 0.009; respectively). The multivariable analyses showed that HbA1c and the presence of DR at baseline were predictive of cardiovascular outcomes (*p* = 0.045 and *p* = 0.023, respectively). However, the burden of subclinical atherosclerosis was not (*p* = 0.783 and *p* = 0.071, respectively).

**Conclusion:**

DR is a strong predictor of cardiovascular events in T2D individuals at primary CVD prevention, even after accounting for the presence of preclinical carotid atherosclerosis. These results may help to individualize CVD prevention strategies in T2D.

## Introduction

Type 2 diabetes mellitus is associated with a higher risk of cardiovascular disease (CVD) (1). The most common clinical expression of CVD is coronary heart disease, cerebrovascular disease, peripheral artery disease, and congestive heart failure (2). These are often manifested as major adverse cardiovascular events with hospitalizations, procedures, and deaths derived from acute coronary syndromes, myocardial infarction, and stroke, as well as sudden death (1, 2). According to the American Diabetes Association (ADA), diabetic retinopathy (DR) is the most common microvascular and neurovascular complication (3). DR is an independent predictor of subclinical CVD in type 2 diabetes (T2D) (4). Furthermore, DR is associated with CVD in patients with T2D (5). Similarly, subclinical atherosclerosis is an independent predictor of cardiovascular events and death in subjects with type 2 diabetes (6, 7). Besides, DR alone or in combination with other microvascular complications has been independently associated with the presence of carotid atherosclerosis in this population (8–10).

A significant number of studies were performed to assess the relative risk of CVD with the presence of DR in subjects with diabetes (11–29). Retrospective studies conducted with large T2D samples found that the CVD risk increases with the cumulative burden of microvascular complications, i.e., DR, diabetic nephropathy, and neuropathy (11, 13). Furthermore, a recent prospective study determined that incident myocardial infarction and stroke events were associated with the presence and severity of DR in this population (12). The World Health Organization (WHO) multinational study group reported that DR is positively associated with cardiovascular mortality and incident myocardial infarction in individuals with T2D (22). Moreover, other studies found that subjects with T2D with a mild or moderate DR had a higher risk of coronary heart disease, stroke, and any CVD (17, 18, 26, 27). A meta-analysis of observational studies found a higher risk of heart failure and stroke in individuals with DR (16). In addition, a prospective study performed with individuals with T2D found that DR was a predictor of cardiovascular events independently of classical risk factors (21). Moreover, a recent retrospective cohort study performed with Catalan population found that DR was associated with coronary heart disease, cardiovascular events, and all-cause mortality among subjects with T2D (20).

Furthermore, a recent meta-analysis reported that DR increases the risk of cardiovascular death in patients with diabetes (30). However, a *post hoc* analysis from a randomized clinical trial did not show any association between DR and increased risk of recurrent cardiovascular events in subjects with T2D (14). Nevertheless, other *post hoc* analyses from two cardiovascular outcome clinical trials found an increased risk of CVD associated with microvascular complications (15).

On the other hand, subclinical atherosclerosis has been associated with a higher risk of CVD in subjects with T2D (6, 7, 31, 32). Specifically, carotid plaque calcification predicted cardiovascular events in T2D subjects with a low degree of stenosis (6). Furthermore, carotid plaques were predictors of cardiovascular morbidity in individuals with T2D without prior CVD (7). Moreover, the presence of carotid plaques was more predictive for prevalent silent coronary atherosclerosis, severity, and extent in asymptomatic T2D individuals (31); besides, intima-media thickness (IMT) and carotid plaques were independent predictors of CVD (32). However, the role of subclinical atherosclerosis in CVD risk assessment is still unclear (33).

To our knowledge, there are no reports investigating together in the same study the potential predictor role of DR and subclinical atherosclerosis on the development of cardiovascular events in subjects with T2D. We hypothesized that as the presence of subclinical atherosclerosis involves detecting the etiological process leading to atherosclerotic cardiovascular events, DR may contribute to further predicting CVD in type 2 diabetes. Therefore, we aimed to assess the predictive role of subclinical atherosclerosis and DR on the development of cardiovascular outcomes in subjects with T2D without prior CVD without chronic kidney disease.

## Materials and methods

Two cohorts of individuals with T2D from the same geographical area recruited in two previous studies were included (10, 34). These subjects were prospectively followed to determine incident cardiovascular events. The first cohort (Cohort 1) was previously selected to study the relationship between carotid atherosclerosis and diabetic retinopathy in T2D. From 312 subjects recruited in this cohort, we included 310 subjects with complete eye and carotid ultrasound assessments from this cohort. The characteristics of the individuals were detailed in a previous publication (10). Briefly, the inclusion criteria in this study were as follows: diagnosis of T2D; age from 40 to 75 years; absence of a prior history of CVD; absence of impaired renal function defined as a estimated glomerular filtration rate (eGFR) < 60 ml/min or macroalbuminuria; no previous history of diabetic foot disease; absence of major psychiatric disorders. The second cohort (Cohort 2) was recruited to investigate the frequency of preclinical carotid atherosclerosis (the DIABIMCAP Study) (34). From a cohort of 106 subjects with T2D included in the DIABIMCAP Study, a total sample of 64 subjects with eye and carotid ultrasound data were included in the current study. A detailed description of this cohort can be found elsewhere (34). The inclusion criteria were a diagnosis of T2D within the previous year; the absence of prior CVD, cancer, chronic renal or liver disease; history of alcohol or drug abuse; major psychiatric or chronic illness, and short life expectancy. Therefore, a final sample of 374 individuals with T2D was prospectively followed. Written informed consent was obtained from each patient included in the study. The study protocol conforms to the ethical guidelines of the 1975 Declaration of Helsinki, and the study has been priorly approved by both Institution’s ethics committees on research on humans.

### Clinical variables

A description of the procedures to collect clinical variables of the two cohorts has been previously published (10, 34). Age, sex, tobacco exposure (current or former smoking), systolic blood pressure (sBP), diastolic blood pressure (dBP), and diabetes duration were collected. Hypertension and dyslipidemia were defined as active treatment with antihypertensive and lipid-lowering agents, respectively. Weight, height, waist, and blood pressure were measured by standard procedures. Blood and urine samples were collected in the fasting state to obtain biochemical parameters using standardized methods. The estimated glomerular filtration rate (eGFR) was calculated using The Modification of Diet in Renal Disease (MDRD-4) formula (35). A thorough revision of the medical records, including all the available information from the health system was performed to identify death and its causes and cardiovascular events during the follow-up period; these included ischemic heart disease (including any recorded diagnosis of any form of ischemic heart disease, and angina pectoris), stroke, heart failure, peripheral artery disease, procedures of revascularization, and cardiovascular mortality. The cardiovascular events were only confirmed when the treating physician had recorded a new diagnosis corresponding to that given event after hospital admission or, alternatively, in the outpatient medical records.

### Diabetic retinopathy

The study participants underwent a complete baseline examination by an ophthalmologist. According to the international consensus on DR (36), DR was assessed with a multifield stereoscopic retinal photography. Moreover, this was classified as follows: (a) no DR; (b) mild non-proliferative DR if only microaneurysms were observed; (c) moderate non-proliferative DR if more than just microaneurysms but less than severe non-proliferative DR were detected; (d) severe non-proliferative DR if any of the following characteristics were observed: over 20 intraretinal hemorrhages in each of 4 quadrants, definite venous beading in 2 + quadrants, prominent intraretinal microvascular abnormalities in 1 + quadrant, as well as no signs of proliferative DR; and (e) proliferative DR if neovascularization and/or vitreous/preretinal hemorrhage was detected (10, 34).

### Carotid ultrasound imaging

All the study subjects underwent the same carotid ultrasound imaging protocol. Bilateral carotid artery B-mode ultrasound imaging to assess IMT and the presence of atherosclerotic plaques was performed following the standardized protocol (10, 34). A semiautomatic software measured IMTs of the common carotid artery (CCA), bulb, and internal carotid artery (ICA). IMT-mean and IMT-maximum from each carotid segment were collected. Atherosclerotic plaques were assessed by using B-mode and color Doppler; these were defined as a focal wall thickening encroaching into the arterial lumen by at least 50% of the surrounding IMT value or with a thickness of 1.5 mm at minimum measuring from the media adventitia interference to the intima-lumen surface, following the Mannheim consensus (37).

### Statistical methods

Continuous variables were tested for normality using the Shapiro–Wilk test. Data are presented as median and 25th and 75th percentiles for continuous variables with a non-normal distribution or the mean and standard deviation (SDs) for variables with a normal distribution. Categorical variables are reported as percentages. Differences between groups were analyzed using the non-parametric Mann–Whitney *U*-test, Welch’s parametric *t*-test for continuous variables, and the chi-square test or Fisher’s exact test for categorical variables. All continuous variables were standardized and normalized whether necessary.

We included subjects with T2D from two different cohorts in the current study. As these patients harbor a different baseline risk, we could not perform Kaplan-Meier and Cox regressions, as they are not appropriate to estimate the survival function in this setting. Therefore, we carried out a series of multivariate predictive models to assess the relationship between prevalent plaque and retinopathy and the cardiovascular outcome. As for the exposure, we divided plaque into Yes (1 or more plaques) and No, or as Multiple plaques (two or more) or Advanced atherosclerosis (three or more plaques). Moreover, DR was classified into either Yes or No, or into No or Mild vs. Severe. For each set of models, we took a dual approach to assess the effect of the predictive variables on the target variable. Random forests prove to be more accurate models for such complex scenarios (38) and, as an added advantage, allowed us to assess the relative importance of each variable *via* out-of-bag accuracy. However, the downside is that random forests do not allow to evaluate whether each variable has a protective or harmful effect. For this reason, we used the result of the regularized regression through which we can evaluate the direction of the effect through the negativity or positivity of the coefficient. Furthermore, in the logistic regression models, we imposed a highly constrained regularization that forces model coefficients to be as small as possible, sometimes even 0. Therefore, if two variables were closely associated with each other, the model removes the effect of the least important one. In the random forests, we constrained the ability of the trees to generate branches, thus reducing the effect of colinear variables. In both cases, the models identified the variable of interest as the most important one in explaining the outcome. Furthermore, in the logistic regression models, we imposed a highly constrained regularization that forces model coefficients to be as small as possible, sometimes even 0. Therefore, if two variables were closely associated with each other, the model removes the effect of the least important one. In the random forests, we constrained the ability of the trees to generate branches, thus reducing the effect of colinear variables. In both cases, the models identified the variable of interest as the most contributing one in explaining the outcome. We provide odds ratios for the logistic regressions and estimates for the linear regressions, both with 95% confidence intervals and *p*-values.

Finally, to study further the etiology of our study, we also built a Bayesian Network among the variables using the PC algorithm (39). This algorithm is able to compute causal relationships between variables. This network provides a graphical representation of the conditional independence relationships among all the variables, allowing us to infer causal relationships between predictor and predicted variables.

All statistical analyses were performed using the R software package version 4.0 (40).

## Results

Clinical characteristics of the study subjects are shown in Table 1. After a median (SD) follow-up of 7.1 (1.0) years, among the 374 participants, 46 reported cardiovascular events. This represents a 1.2% annual rate of cardiovascular events in this primary prevention cohort. Subjects who reported cardiovascular outcomes had a longer diabetes duration (*p* < 0.001), higher total cholesterol (*p* = 0.030), and glycated hemoglobin (HbA1c) (*p* = 0.023) in comparison with those who remained free of cardiovascular events at the end of the follow-up. Furthermore, they had also a higher prevalence of DR (67.4% vs. 38.4%, *p* = 0.001; respectively) and DR severity (45.7% vs. 21.3%, *p* < 0.001; respectively). In addition, mortality was higher in the group with cardiovascular events (17.4% vs. 2.7%, *p* < 0.001; respectively). The description of the study sample according to the presence of atherosclerotic plaques and DR is shown in Supplementary Table 1. Those participants with plaque(s) were older (*p* < 0.001), had higher sBP (*p* = 0.010) and higher frequency of hypertension (*p* = 0.034), tobacco exposure (*p* < 0.001) compared with individuals without atherosclerosis. On the other hand, individuals with DR had a higher frequency of hypertension (*p* = 0.007), atherosclerotic plaques (*p* = 0.014), deaths (*p* = 0.028), cardiovascular mortality (*p* = 0.030), and cardiovascular events (*p* = 0.001) in comparison with the non-DR group. Moreover, they had higher waist (*p* = 0.003), diabetes duration (*p* < 0.001), sBP (*p* < 0.001), HDL-cholesterol (*p* = 0.021), and albumin/creatinine ratio (*p* < 0.001). The clinical characteristics of both study cohorts are shown in Supplementary Table 2. Participants from Cohort 1 were older (*p* = 0.030) and had a lower frequency of tobacco exposure (*p* < 0.001) in comparison with those from Cohort 2. Furthermore, these participants had a higher diabetes duration (*p* < 0.001), sBP (*p* = 0.032), HbA1c (*p* < 0.001), eGFR (*p* < 0.001), and a higher frequency and severity of DR (*p* < 0.001). However, they showed lower dBP (*p* < 0.001), total cholesterol (*p* = 0.007), LDL-cholesterol (*p* = 0.003), and presence of subclinical carotid atherosclerosis (*p* < 0.001) compared with Cohort 2.

**TABLE 1:** Clinical characteristics of the study group according to incident cardiovascular events.

Characteristics	No CVE *N* = 328	CVE *N* = 46	*p*
Sex (women)	150 (45.7)	26 (56.5)	0.224
Age (years)	60.5 [52.0–67.0]	60.0 [54.0–69.0]	0.366
Hypertension	185 (56.4)	31 (67.4)	0.210
Dyslipidemia	142 (43.3)	27 (58.7)	0.071
Waist (cm)	104.0 [97.0–111.0]	106.0 [99.8–114.0]	0.116
BMI (kg/m^2^)	30.3 [27.7–34.2]	32.0 [28.8–35.3]	0.113
Tobacco exposure	184 (56.1)	26 (56.5)	0.511
Diabetes duration (years)	5.4 [2.0–10.0]	11.0 [5.3–20.0]	<0.001
sBP (mmHg)	137.0 [126.0–148.0]	142.0 [130.0–156.0]	0.051
dBP (mmHg)	78.0 [71.5–86.0]	78.0 [69.0–85.8]	0.471
Total cholesterol (mg/dl)	183.0 [163.0–212.0]	193.0 [174.0–223.0]	0.030
HDL-cholesterol (mg/dl)	48.0 [40.8–58.0]	48.0 [45.0–63.0]	0.086
LDL-cholesterol (mg/dl)	108.0 [88.4–130.0]	112.0 [95.8–133.0]	0.345
Triglycerides (mg/dl)	118.0 [87.0–167.0]	128.0 [81.2–171.0]	0.635
HbA1c (%)	7.3 [6.6–8.3]	7.8 [6.9–9.4]	0.023
Albumin/creatinine ratio (mg/g)	7.0 [3.9–14.7]	12.0 [5.0–25.0]	0.027
eGFR (mL/min/1.73 m^2^)	90.4 [77.7–104.0]	91.1 [85.4–104.0]	0.376
Subclinical carotid atherosclerosis	203 (61.9)	32 (69.6)	0.538
Categorized subclinical carotid aterosclerosis			0.188
No	125 (38.1)	14 (30.4)	
One plaque	86 (26.2)	8 (17.4)	
Multiple plaques	117 (35.7)	24 (52.2)	
Advanced aterosclerosis	60 (18.2)	15 (34.1)	0.014
Diabetic retinopathy	126 (38.4)	31 (67.4)	0.001
DR status			0.001
No	202 (61.6)	15 (32.6)	
Mild	56 (17.1)	10 (21.7)	
Moderate or severe	70 (21.3)	21 (45.7)	
Deaths	9 (2.7)	8 (17.4)	<0.001
Cardiovascular mortality	0 (0.0)	3 (6.5)	0.002
Ischemic heart disease	0 (0.0)	24 (52.2)	<0.001
Stroke	0 (0.0)	9 (19.6)	<0.001
Heart failure	0 (0.0)	13 (28.3)	<0.001
Peripheral artery disease	0 (0.0)	11 (23.9)	<0.001
Procedure of revascularization	0 (0.0)	12 (26.1)	<0.001

Data are shown as n (%) for categorical variables and median [interquartile range] for continuous variables. Multiple plaques include two or more plaques. Advanced atherosclerosis consists of the presence of three or more plaques. CVE, cardiovascular events; DR, diabetic retinopathy; dBP, diastolic blood pressure; BMI, body mass index; eGFR, estimated glomerular filtration rate; HbA1c, glycated hemoglobin; HDL-cholesterol, high-density lipoprotein-cholesterol; LDL-cholesterol, low-density lipoprotein-cholesterol; sBP, systolic blood pressure.

### Diabetic retinopathy, preclinical atherosclerosis, and cardiovascular events

**FIGURE 1:**
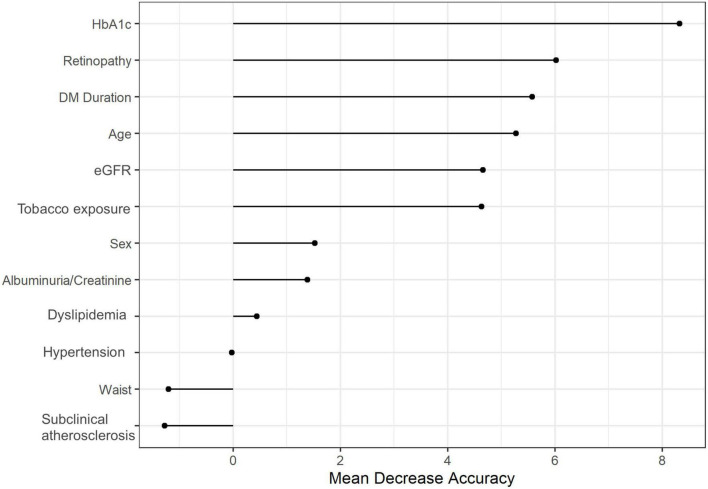
Random forest of the relative importance of each variable in the multivariate model for the association between the presence of atherosclerotic plaque and diabetic retinopathy with cardiovascular events. HbA1c, glycated hemoglobin; DM_duration, diabetes mellitus duration; Plaque, presence of carotid plaques; eGFR, estimated glomerular filtration rate.

The multivariate predictive model for cardiovascular events showed that both HbA1c (1% change) and the presence of DR were predictors of cardiovascular events [OR 1.09, 95% CI (1.00–1.18), *p* = 0.045 and OR 1.27, 95% CI (1.06–1.52), *p* = 0.023; respectively] after accounting for confounders (Table 2). However, the presence of subclinical carotid atherosclerotic plaques was not associated with cardiovascular events [OR 1.06, 95% CI (0.90–1.25), *p* = 0.833]. In the relative variable importance of this model, HbA1c and DR had a higher mean decrease accuracy being the two most important variables of this model (Figure 1).

**TABLE 2:** Regularized logistic regression models for the association between the different variables, including plaque and diabetic retinopathy, and incident major adverse cardiovascular events.

Variables	OR (95% CI)	*p*	OR (95% CI)	*P*
Intercept	0.09 (0.07–0.12)	<0.001	0.10 (0.08–0.12)	<0.001
Sex (women)	1.11 (0.93–1.34)	0.107	1.11 (0.94–1.31)	0.138
Age (years)	1.03 (0.94–1.12)	0.470	1.03 (0.95–1.11)	0.449
Hypertension	1.12 (0.96–1.34)	0.265	1.14 (0.98–1.32)	0.254
Dyslipidemia	1.12 (0.97–1.30)	0.390	1.13 (0.99–1.29)	0.339
Diabetes duration (years)	1.08 (0.98–1.19)	0.439	1.08 (0.99–1.18)	0.323
Waist (cm)	1.06 (0.98–1.14)	0.506	1.06 (0.99–1.13)	0.539
Tobacco exposure	1.05 (0.86–1.27)	0.124	1.05 (0.88–1.25)	0.132
HbA1c (%)	1.09 (1.00–1.18)	**0.045**	1.09 (1.02–1.17)	**0.034**
Albumin/ creatinine ratio (mg/g)	1.06 (0.98–1.14)	0.870	1.06 (0.99–1.13)	0.815
eGFR (mL/min/1.73 m^2^)	1.03 (0.95–1.11)	0.386	1.03 (0.96–1.10)	0.338
Subclinical carotid atherosclerosis	1.06 (0.90–1.25)	0.833	1.06 (0.92–1.23)	0.783
Diabetic retinopathy	1.27 (1.06–1.52)	**0.023**	-	-
Diabetic retinopathy (severe)	-	-	1.27 (0.92–1.76)	0.074

AUC = 0.69 for the model including the presence of subclinical atherosclerosis and retinopathy. AUC = 0.68 for the model including the presence of subclinical atherosclerosis and severity of retinopathy. Severe diabetic retinopathy group compared with the group with mild or no DR. CI, confidence interval; eGFR, estimated glomerular filtration rate; HbA1c, glycated hemoglobin; OR, odds ratio. The bold values are statistical significants with p values lower than 0.05.

**FIGURE 2:**
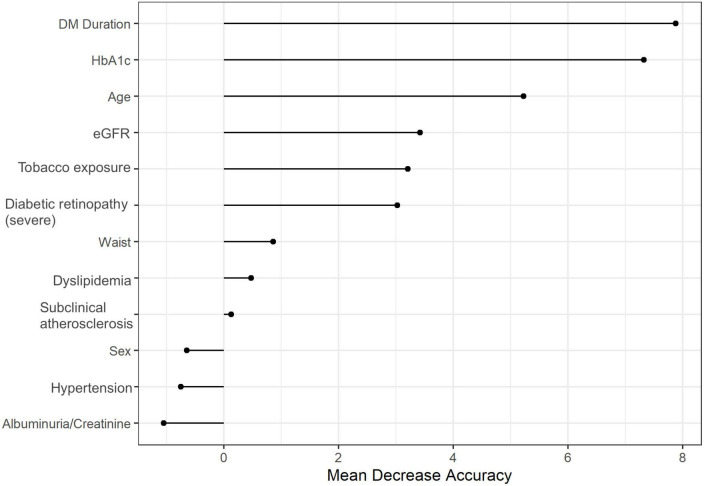
Random forest of the relative importance of each variable in the multivariate predictive model for the association between severe diabetic retinopathy and the presence of subclinical atherosclerosis with cardiovascular events. DM_duration, diabetes mellitus duration; eGFR, estimated glomerular filtration rate; Plaque, presence of carotid plaques. Diabetic retinopathy severe, reference group: no or mild diabetic retinopathy.

On the other hand, when considering in this model a more advanced definition of DR, preclinical atherosclerosis did not have a predictive role in the development of cardiovascular events either [OR 1.06, 95% CI (0.92–1.23); *p* = 0.783] (Table 2). In this case, severe DR was not significantly associated with cardiovascular events [OR 1.27, 95% CI (0.92–1.76); *p* = 0.074]. Besides, diabetes duration and HbA1c had a decisive role in this multivariate prediction model, as shown in Figure 2. Nevertheless, HbA1c had a risk association of cardiovascular events [OR 1.09, 95% CI (1.02–1.17), *p* = 0.034]. Furthermore, the presence of DR or advanced atherosclerosis (three or greater plaques) were not predictive factors of cardiovascular events [OR 1.41 95% CI (1.12–1.77); *p* = 0.091, and OR 1.44 (1.16–1.78); *p* = 0.071, respectively] in multivariate models (Supplementary Table 3).

### Causal inference on cardiovascular events

**FIGURE 3:**
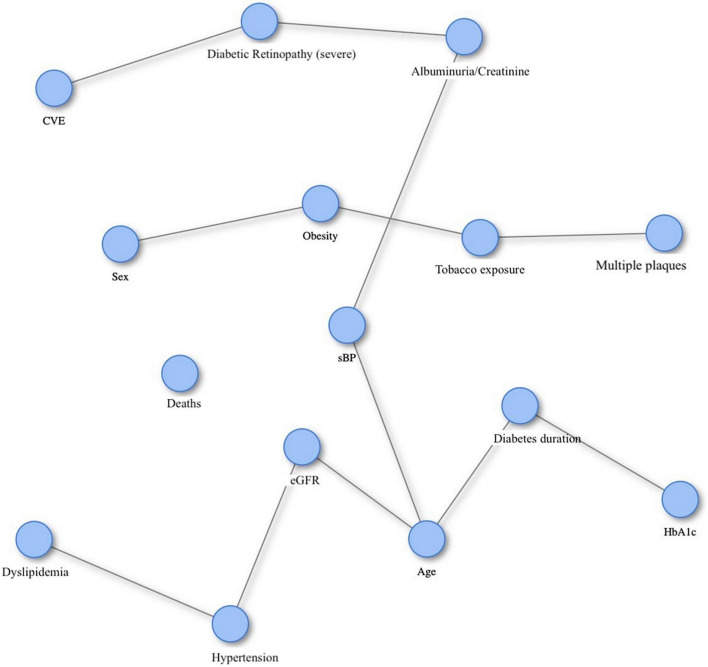
The prediction causation algorithm for the causal relationship between diabetic retinopathy, subclinical atherosclerosis and cardiovascular events in subjects with type 2 diabetes. CVE, cardiovascular events; Multiple plaques, multiple carotid plaques (two or more); sBP, systolic blood pressure; eGFR, estimated glomerular filtration rate; DM_duration, diabetes mellitus duration; HbA1c, glycated hemoglobin.

The prediction causation algorithm to explore the causal relationship between DR, subclinical carotid atherosclerosis and cardiovascular outcomes showed that severe DR had a causal solid connection for the development of cardiovascular events (Figure 3). Subclinical atherosclerosis was not related to cardiovascular events in our study sample. Nevertheless, female sex, obesity, and tobacco exposure were associated with the development of severe atherosclerosis, defined as the presence of three or more plaques. In addition, all-cause mortality was not associated with any of the other clinical variables even though, as expected, classical cardiovascular risk factors were related to the increasing albumin/creatinine ratio and, consequently, the presence of severe DR.

## Discussion

This is the first study that assessed the independent predictive role of DR and subclinical atherosclerosis in developing cardiovascular events in T2D individuals without a history of the previous CVD at baseline and established chronic kidney disease. Our findings showed that HbA1c and DR, but not preclinical atherosclerosis, were predictors of cardiovascular events considering the presence of subclinical atherosclerosis.

The presence of DR and a poorer glycemic control were associated with a higher risk of developing a cardiovascular event in subjects with T2D, adjusting for classical risk factors and considering the presence and burden of subclinical atherosclerosis; this is in line with previous studies (20, 21). The burden of microvascular complications such as DR, nephropathy, and neuropathy was associated with a cumulative risk of cardiovascular death, non-fatal myocardial infarction, or non-fatal ischemic stroke in subjects with T2D (11). Furthermore, microvascular complications were independently related to the 10-year risk of death and cardiovascular events in individuals with T2D (13). A recent systematic review reported that DR is a strong predictor of stroke and CVD, suggesting that patients with DR have an overall worse prognosis than those without this condition (5). On the other hand, a *post hoc* analysis from a clinical trial did not find any association between DR or neuropathy with cardiovascular death, non-fatal myocardial infarction, stroke, hospitalization for unstable angina, and recent acute coronary syndrome (14). Nevertheless, the authors identified the diabetes duration as an independent predictor of cardiovascular events beyond DR, neuropathy, and cardiovascular risk factors, which is in line with our findings on the importance of the disease duration in the predictive models. In addition, the WHO study researchers found that diabetes duration and glycemic control predicted incident fatal and non-fatal myocardial infarction in subjects with diabetes (22). Therefore, the role of DR, glycemic control, and diabetes duration in predicting cardiovascular events should be considered in this population. The potential relationship between DR and subclinical atherosclerosis has been published in previous studies (8–10, 41); additionally, the American Heart Association (AHA) and the European Society of Cardiology (ESC) included DR as an independent risk factor to predict cardiovascular events in patients treated in primary prevention (42, 43).

On the other hand, subclinical atherosclerosis did not predict a higher risk of developing cardiovascular events in subjects with T2D; however, the high prevalence (63%) of patients with subclinical atherosclerosis in our study sample may be a limitation to assess its predictive role, as well as that of other highly prevalent classical risk factors such as age, dyslipidemia, and hypertension. The presence of atherosclerotic plaques predicts future cardiovascular events and mortality in patients with T2D (6, 7), a finding that is in contrast with our results. However, participants in previously published studies had other late diabetic complications, such as nephropathy, that could strongly influence the outcome. Individuals with a higher burden of plaques have a higher incidence of renal endpoints (7); moreover, carotid plaques are predictors of renal outcomes in subjects with T2D. Nevertheless, microvascular complications and subclinical atherosclerosis may be closely linked in their respective pathogenetic pathways (19). Our findings identify DR as a causal predicting factor in developing a cardiovascular events in the prediction causation algorithm; however, this cannot be definitively established because microangiopathy and carotid atherosclerosis are closely linked (8).

One of the limitations of our study is that we used two different cohorts to establish the predictive role of DR and subclinical atherosclerosis in the development of cardiovascular events. However, regularized logistic regression models were performed to calculate the study participants’ risk. Although the causal relationships between cardiovascular events and predictors were calculated using a Bayesian method, we cannot conclude this causal relationship due to the study design. Moreover, definitive conclusions cannot be established due to the rather insufficient number of incident cardiovascular events and the short follow-up study period of the participants; for this reason, the burden of subclinical atherosclerosis and severe DR, as well as the other classical risk factors such as age, dyslipidemia, and hypertension did not have either a significant association with cardiovascular events. Moreover, classical risk factors such as hypertension and dyslipidemia could be associated with each other which could influence the results. Furthermore, any association of cardiovascular events with HbA1c and DR would have not been found if the traditional definition of major adverse cardiovascular events (MACE, i.e., non-fatal myocardial infarction, non-fatal stroke and cardiovascular death) were used as the total number of events would have been even lower. In addition, data from study subjects on their dietary pattern, physical activity and alcohol consumption were not available. Therefore, their potential contribution of these factors to the main outcome of the study could not be included in the models. On the other hand, this is the first prospective study that analyzed the predictive role of DR and subclinical atherosclerosis on the cardiovascular events. Therefore, these results may be highly relevant for the design of further studies focused on this issue. Furthermore, this study included a well-defined sample including participants without other diabetic complications and no history of previous CVD, allowing us specifically assessing the predictive role of DR and subclinical atherosclerosis.

## Conclusion

In conclusion, the presence of DR was a strong predictor of CVD independently from the presence of subclinical carotid atherosclerosis in subjects with T2D without previous clinical CVD. Our findings point to a solid linkage between DR and future CVD in T2D, which aligns with the published studies and guidelines in this issue. However, more extensive studies are needed to confirm the current findings.

## Data availability statement

The raw data supporting the conclusions of this article will be made available by the authors, without undue reservation.

## Ethics statement

The studies involving human participants were reviewed and approved by the University Hospital Arnau de Vilanova, Lleida, Spain (CEIC 7/2011) and University Hospital Clinic de Barcelona, Barcelona, Spain (CEIC 2011/6466). The patients/participants provided their written informed consent to participate in this study.

## Author contributions

EC, MG-C, EO, and DM contributed to the conception and design of the study. EC, MG-C, MH, MP, JJ, MR-L, and NA conducted the study. ECo analyzed the data. EC, MG-C, AA, EO, and DM interpreted the results. EC and MG-C wrote the manuscript. EO and DM were guarantors of this work and as such had full access to all the data in the study and take full responsibility for the integrity of the data, and the accuracy of the data analysis. All authors have revised the manuscript critically for important intellectual content and given final approval of the version to be published.
