# Supplementary material for: Diabetic retinopathy predicts cardiovascular disease independently of subclinical atherosclerosis in individuals with type 2 diabetes: A prospective cohort study
Source: Front Cardiovasc Med. 2022 Nov 3;9:945421. doi: 10.3389/fcvm.2022.945421 (PMC9668891; doi:10.3389/fcvm.2022.945421)
Supplement: Supplementary file 1 [file Data_Sheet_1.docx]

Supplementary Material

**Table S1.** Descriptive analysis of participants according to the presence of plaques and diabetic retinopathy.

| **Characteristics** | **No plaque**  **N = 139** | **Plaque(s)**  **N = 235** | **p** | **No DR**  **N = 217** | **DR**  **N = 157** | **p** |
| --- | --- | --- | --- | --- | --- | --- |
| Sex, women | 73 (52.5) | 103 (43.8) | 0.129 | 98 (45.2) | 78 (49.7) | 0.448 |
| Age, years | 58.0 [48.5;64.0] | 62.0 [55.0;68.5] | <0.001 | 60.0 [51.0;66.0] | 61.0 [54.0;68.0] | 0.056 |
| Hypertension | 70 (50.4) | 146 (62.1) | 0.034 | 112 (51.6) | 104 (66.2) | 0.007 |
| Dyslipidemia | 58 (41.7) | 111 (47.2) | 0.354 | 91 (41.9) | 78 (49.7) | 0.168 |
| Waist, cm | 104.0 [98.0;112.0] | 104.0 [97.8;111.0] | 0.746 | 103.0 [96.0;110.0] | 106.0 [101.0;113.0] | 0.003 |
| Tobacco exposure | 60 (43.2) | 151 (64.3) | <0.001 | 132 (60.8) | 79 (50.3) | 0.055 |
| Diabetes duration, years | 6.0 [2.0,11.0] | 6.0 [1.0;11.0] | 0.463 | 4.0 [1.0;8.0] | 10.0 [5.0;20.0] | <0.001 |
| sBP | 136.0 [123.0;146.0] | 139.0 [128.0;150.0] | 0.010 | 134.0 [123.0;145.0] | 143.0 [132.0;158.0] | <0.001 |
| dBP | 78.0 [72.0;86.5] | 78.0 [70.0;85.0] | 0.415 | 79.0 [72.0]86.0] | 78.0 [69.0;86.0] | 0.250 |
| Total cholesterol, mg/dl | 185.0 [163.0;214.0] | 186.0 [164.0;212.0] | 0.842 | 186.0 [165.0;215.0] | 181.5 [163.0;212.5] | 0.480 |
| HDL-cholesterol, mg/dl | 46.0 [40.0;57.0] | 48.0 [42.0;60.0] | 0.173 | 47.0 [40.0;56.0] | 50.5 [42.0;60.5] | 0.021 |
| LDL-cholesterol, mg/dl | 108.6 [87.2;131.6] | 109.0 [91.0;130.0] | 0.890 | 113.0 [91.8;132.0] | 106.0 [86.5;128.2] | 0.061 |
| Triglycerides, mg/dl | 122.0 [89.0;176.0] | 116.0 [85.0;165.0] | 0.235 | 120.0 [89.0;172.0] | 117.0 [84.0;168.0] | 0.490 |
| HbA1c, % | 7.4 [6.6;8.6] | 7.3 [6.6;8.2] | 0.610 | 6.9 [6.4;7.8] | 8.1 [7.2;9.1] | <0.001 |
| Albumin/creatinine ratio | 6.9 [3.3,15.2] | 7.7 [4.0;17.5] | 0.214 | 5.9 [3.3;11.0] | 11.6 [5.7;32.1] | <0.001 |
| eGFR, mL/min/1.73m^2^ | 93.4 [80.1;105.0] | 89.7 [77.9;104.0] | 0.256 | 89.7 [78.6;104.0] | 91.0 [80.0;103.0] | 0.723 |
| Subclinical carotid atherosclerosis | - | - | - | 128 (59.0) | 107 (68.2) | 0.089 |
| Categorized subclinical carotid atherosclerosis |  |  | <0.001 |  |  | 0.014 |
| None or one | 139 (100.0) | 94 (40.0) |  | 147 (67.7) | 86 (54.8) |  |
| Multiple plaques | 0 (0.0) | 141 (60.0) |  | 70 (32.3) | 71 (45.2) |  |
| Diabetic retinopathy | 50 (36.0) | 107 (45.5) | 0.089 | - | - | - |
| DR status |  |  | 0.118 |  |  | <0.001 |
| No | 89 (64.0) | 128 (54.5) |  | 217 (100.0) | 0 (0.0) |  |
| Mild | 24 (17.3) | 42 (17.9) |  | 0 (0.0) | 66 (42.0) |  |
| Moderate or severe | 26 (18.7) | 65 (27.7) |  | 0 (0.0) | 91 (58.0) |  |
| Deaths | 4 (2.9) | 13 (5.5) | 0.350 | 5 (2.3) | 12 (7.6) | 0.028 |
| Cardiovascular mortality | 0 (0.0) | 3 (1.3) | 1.000 | 0 (0.0) | 3 (1.9) | 0.030 |
| Cardiovascular events | 14 (10.1) | 30 (12.8) | 0.538 | 15 (6.9) | 29 (18.5) | 0.001 |

Data are shown as n (%) for categorical variables and as median [interquartile range] for continuous variables. Multiple plaques include two or more plaques. DR, diabetic retinopathy; dBP, diastolic blood pressure; eGFR, estimated glomerular filtration rate; HbA1c, glycated hemoglobin; HDL-cholesterol, high density lipoprotein-cholesterol; LDL-cholesterol, low density lipoprotein-cholesterol; MACE, major adverse cardiovascular event; sBP, systolic blood pressure.

**Table S2.** Clinical characteristics of the study cohorts

| **Characteristics** | **Cohort 1**  **N = 310** | **Cohort 2**  **N = 64** | **p** |
| --- | --- | --- | --- |
| Sex (women) | 153 (49.4) | 23 (35.9) | 0.069 |
| Age (years) | 60.0 [51.0;67.0] | 63.0 [58.8;66.0] | **0.030** |
| Hypertension | 181 (58.4) | 35 (54.7) | 0.684 |
| Dyslipidemia | 144 (46.5) | 25 (39.1) | 0.345 |
| Waist (cm) | 104.0 [98.0;112.0] | 102.0 [95.4;109.0] | 0.051 |
| BMI (kg/m^2^) | 30.6 [28.0;34.7] | 29.9 [27.1;32.8] | 0.080 |
| Tobacco exposure | 160 (51.6) | 50 (78.1) | **<0.001** |
| Diabetes duration (years) | 8.0 [4.0;14.0] | 0.2 [0.1;0.6] | **<0.001** |
| Follow-up (years) | 7.2 [6.5;7.9] | 7.1 [6.6;7.5] | 0.057 |
| sBP (mmHg) | 139.0 [127.0;150.0] | 134.0 [124.0;145.0] | **0.032** |
| dBP (mmHg) | 77.0 [70.0;84.0] | 84.0 [76.8;89.2] | **<0.001** |
| Total cholesterol (mg/dl) | 182.0 [163.0;206.0] | 198.0 [172.0;232.0] | **0.007** |
| HDL-cholesterol (mg/dl) | 48.0 [41.0;59.0] | 46.5 [40.8;53.2] | 0.476 |
| LDL-cholesterol (mg/dl) | 107.0 [87.6;129.0] | 116.0 [102.0;149.0] | **0.003** |
| Triglycerides (mg/dl) | 118.0 [85.0;168.0] | 126.0 [89.0;171.0] | 0.333 |
| HbA1c (%) | 7.5 [6.8;8.4] | 6.8 [6.4;7.5] | **<0.001** |
| Albumin/creatinine ratio (mg/g) | 8.1 [3.8;18.0] | 6.0 [4.0;10.8] | 0.127 |
| eGFR (mL/min/1.73m^2^) | 92.3 [82.0;105.0] | 83.2 [73.9;89.5] | **<0.001** |
| Subclinical carotid atherosclerosis | 185 (59.7) | 50 (78.1) | **<0.001** |
| Categorized subclinical carotid atherosclerosis |  |  | **<0.001** |
| No | 125 (40.3) | 14 (21.9) |  |
| One plaque | 85 (27.4) | 9 (14.1) |  |
| Multiple plaques | 100 (32.3) | 41 (64.1) |  |
| Diabetic retinopathy | 151 (48.7) | 6 (9.4) | **<0.001** |
| DR status |  |  | **<0.001** |
| No | 159 (51.3) | 58 (90.6) |  |
| Mild | 60 (19.4) | 6 (9.4) |  |
| Moderate or severe | 91 (29.4) | 0 (0.0) |  |
| Deaths | 15 (4.8) | 2 (3.1) | 0.748 |
| Cardiovascular mortality | 3 (0.9) | 0 (0.0) | 1.000 |
| Cardiovascular events | 41 (13.2) | 5 (7.8) |  |

Data are shown as n (%) for categorical variables and as median [interquartile range] for continuous variables. Multiple plaques include two or more plaques. DR, diabetic retinopathy; dBP, diastolic blood pressure; eGFR, estimated glomerular filtration rate; HbA1c, glycated hemoglobin; HDL-cholesterol, high density lipoprotein-cholesterol; LDL-cholesterol, low density lipoprotein-cholesterol; MACE, major adverse cardiovascular event; sBP, systolic blood pressure.

**Table S3.** Regularized logistic predicted model for the association between the presence of diabetic retinopathy, advanced atherosclerosis, and incident major adverse cardiovascular events.

| **Variables** | **OR (95% CI)** | **p** |
| --- | --- | --- |
| Intercept | 0.07 (0.05-0.10) | <0.001 |
| Sex, women | 1.21 (0.95-1.52) | 0.091 |
| Age, years | 1.03 (0.92-1.16) | 0.783 |
| Hypertension | 1.19 (0.96-1.47) | 0.373 |
| Dyslipidemia | 1.17 (0.96-1.41) | 0.553 |
| Diabetes duration, years | 1.12 (0.99-1.27) | 0.338 |
| Waist, cm | 1.08 (0.98-1.19) | 0.448 |
| Tobacco exposure | 1.10 (0.86-1.40) | 0.187 |
| HbA1c, % | 1.13 (1.02-1.24) | 0.370 |
| Albuminuria/Creatinine | 1.07 (0.97-1.18) | 0.791 |
| eGFR, mL/min/1.73m^2^ | 1.05 (0.95-1.15) | 0.523 |
| Advanced atherosclerosis | 1.44 (1.16-1.78) | 0.071 |
| Diabetic retinopathy | 1.41 (1.12-1.77) | 0.091 |

Advanced atherosclerosis consists of the presence of three or more plaques. AUC = 0.71.

HbA1c, glycated hemoglobin; eGFR, estimated glomerular filtration rate.
